# Generation of *Pet1_210_-Cre* Transgenic Mouse Line Reveals Non-Serotonergic Expression Domains of *Pet1* Both in CNS and Periphery

**DOI:** 10.1371/journal.pone.0104318

**Published:** 2014-08-06

**Authors:** Barbara Pelosi, Sara Migliarini, Giulia Pacini, Marta Pratelli, Massimo Pasqualetti

**Affiliations:** 1 Department of Biology, Unit of Cell and Developmental Biology, University of Pisa, Pisa, Italy; 2 Istituto Italiano di Tecnologia, Center for Neuroscience and Cognitive Systems @UniTn, Rovereto, Italy; IGBMC/ICS, France

## Abstract

Neurons producing serotonin (5-hydroxytryptamine, 5-HT) constitute one of the most widely distributed neuronal networks in the mammalian central nervous system (CNS) and exhibit a profuse innervation throughout the CNS already at early stages of development. Serotonergic neuron specification is controlled by a combination of secreted molecules and transcription factors such as Shh, Fgf4/8, Nkx2.2, Lmx1b and Pet1. In the mouse, *Pet1* mRNA expression appears between 10 and 11 *days post coitum (*dpc) in serotonergic post-mitotic precursors and persists in serotonergic neurons up to adulthood, where it promotes the expression of genes defining the mature serotonergic phenotype such as *tryptophan hydroxylase 2* (*Tph2*) and *serotonin transporter* (*SERT*). Hence, the generation of genetic tools based on *Pet1* specific expression represents a valuable approach to study the development and function of the serotonergic system. Here, we report the generation of a *Pet1_210_-Cre* transgenic mouse line in which the *Cre recombinase* is expressed under the control of a 210 kb fragment from the *Pet1* genetic locus to ensure a reliable and faithful control of somatic recombination in *Pet1* cell lineage. Besides Cre-mediated recombination accurately occurred in the serotonergic system as expected and according to previous studies, *Pet1_210_-Cre* transgenic mouse line allowed us to identify novel, so far uncharacterized, *Pet1* expression domains. Indeed, we showed that in the *raphe Pet1* is expressed also in a non-serotonergic neuronal population intermingled with *Tph2*-expressing cells and mostly localized in the B8 and B9 nuclei. Moreover, we detected Cre-mediated recombination also in the developing pancreas and in the ureteric bud derivatives of the kidney, where it reflected a specific *Pet1* expression. Thus, *Pet1_210_-Cre* transgenic mouse line faithfully drives Cre-mediated recombination in all *Pet1* expression domains representing a valuable tool to genetically manipulate serotonergic and non-serotonergic *Pet1* cell lineages.

## Introduction

In mammals, neurons producing serotonin (5-hydroxytryptamine, 5-HT) are generated early during embryonic development in the ventral hindbrain and progressively cluster into B1–B9 *raphe* nuclei that project to the whole central nervous system (CNS), from the anterior brain to the spinal cord [1], [2], [3]. The extensive and capillary organization of serotonergic terminals together with the existence of at least 15 different 5-HT receptors distributed in the CNS accounts for the multitude of physiological and behavioural functions mediated by brain serotonin, from the regulation of circadian rhythms [4] and mood [5], to social interaction [6] and sexuality [7]. Moreover, in addition to its function in neurotransmission, growing evidences support a role for serotonin in developmental processes as cellular proliferation, migration, neuronal differentiation and brain circuitry formation [8]–[12]. Consistently, an altered serotonergic signalling has been associated with neuropsychiatric disorders in humans thought to have neurodevelopmental basis, such as schizophrenia and autism [13],[14].

During the last years, growing efforts have been made to generate suitable genetic tools to target serotonergic neurons in order to study their development and function [15]–[18]. Advances in mouse molecular genetics have brought new insights into the comprehension of the molecular cascade involved in serotonergic neuron specification [19]–[24], as well as of the projection network of *raphe* nuclei [11], [25]. Altogether, these studies have been crucial to reconsider serotonergic system, rather than a homogenous ensemble, a complex and heterogeneous population with distinct morphological, molecular and electrophysiological characteristics [22], [24]–[29].

The transcriptional pathways of serotonergic neuron differentiation require a combination of secreted molecules and transcription factors such as Shh, Fgf4/8, Nkx2.2 and Lmx1b, and converge on the activation of the ETS transcription factor Pet1 (plasmacytoma expressed transcription factor 1, official name Fev) [24], [30]. The onset of *Pet1* expression in the mouse has been described to occur approximately as early as 10.5 dpc in post-mitotic precursors within the mantle layer, in the rostral hindbrain from rhombomere (r) 1 to r3, (i.e. rostral serotonergic domain), and one day later in r5-r7 (i.e. caudal cluster) [30]–[32]. In these domains *Pet1* expression precedes the appearance of markers of serotonergic terminal differentiation, such as *tryptophan hydroxylase 2* (*Tph2*) and *serotonin transporter* (*SERT*), and is maintained up to adulthood [30].

Thus, thanks to its early and specific expression, *Pet1* represents the ideal candidate gene to be used in Cre recombinase/loxP-based strategies to specifically target serotonergic neurons. Such an approach represents a powerful tool to map the genetic lineage of *Pet1* expressing cells and to characterize the molecular identity of distinct subpopulations of serotonergic neurons through intersectional strategies [33]. Moreover, a *Cre recombinase*-expressing line targeting serotonergic neurons could be used in conditional knock out approaches to investigate the involvement of specific genes in the development and functioning of serotonergic system, as well as in studies aimed to map serotonergic structural and functional connections in the brain.

In this context, the availability of BAC (Bacterial Artificial Chromosome)-based homologous recombination in *E. coli* approach allows the generation of large transgenic constructs, thus providing the presence of long distance acting regulatory elements required for the proper temporal- and tissue-specific expression of the gene of interest as well as reducing positional effect, that otherwise might drive transgene expression outside promoter-specific cell populations [34]–[39].

In the present study we used an *E. coli* homologous recombination based-approach to generate a *Pet1_210_-Cre* transgenic mouse line, in which 210 kb of *Pet1* locus drive the expression of the *Cre recombinase*. Our analysis showed that in *Pet1_210_-Cre* mice Cre-mediated somatic recombination specifically occurs in serotonergic neurons of the *raphe* nuclei. Moreover, we demonstrated that *Pet1* is expressed also in a population of non-serotonergic neurons within the *raphe* nuclei and in non-neuronal districts such as the ureteric bud derivatives of the kidney and the pancreas, starting from 9.5 dpc and 11.5 dpc, respectively. The *Pet1_210_-Cre* transgenic mouse line thus widens our knowledge on *Pet1* gene expression and represents a valuable tool to promote Cre-mediated somatic recombination both in serotonergic and non-serotonergic *Pet1* cell progeny.

## Materials and Methods

### Animals

Mice were housed in standard Plexiglas cages at constant temperature (22±1°C) and maintained on a 12/12 h light/dark cycle, with food and water *ad libitum*. Experimental protocols were conducted in accordance with the Ethic Committee of the University of Pisa and approved by the Veterinary Department of the Italian Ministry of Health.

### Generation of the *Pet1_210_-Cre* transgenic mouse line

To generate the BAC-Cre construct we took advantage of a recombination-based strategy carried out in bacteria. To this aim we obtained a *Pet1_210_-Cre* targeting vector containing both the *Cre recombinase* cDNA and a *kana/Neo* resistance gene flanked by two 500 bp long homology arms. Briefly, *Pet1_210_-Cre* left arm (*Pet1_210_LA*) has been cloned in frame with *Cre recombinase* using an overlapping Polymerase Chain Reaction (PCR)-based strategy [40]. Briefly, *Pet1_210_LA* and *Cre recombinase* have been separately amplified by means of PCR using BAC_RP23_165D11 and a pSG5-Cre plasmid as templates, respectively. Amplification of *Pet1_210_LA* was performed using the following primers: forward 5′ ATTATT**CTCGAG**GGGAGGTAGAAAAAGACGCACGTA 3′, reverse 5′ TTGGTGTACGGTCAGTAAATTGGACAT CGCTGCCGGGGACTGGGC 3′. *Cre recombinase* cDNA was amplified using the following primers: forward 5′ GCCCAGTCCCCGGCAGCG ATGTCCAATTTACTGACCGTACACCAA 3′, reverse 5′ ATTATT**CTCGAG**CAGACAATGATAAGATACATTGATGAGTTT 3′. Overlapping sequences for *Pet1_210_LA* and *Cre recombinase* are underlined, and the XhoI site is shown in bold. The amplified fragments were used simultaneously in a second round of PCR using the forward primer of *Pet1_210_LA* and the reverse primer of *Cre recombinase* to obtain a 1.7 kb fragment in which the second codon of *Cre recombinase* is in frame with the first ATG codon of *Pet1* gene. A *kana/Neo* resistance cassette flanked by two FRT sites for Flp recombinase-mediated excision obtained from a pSVKeoX1FRT plasmid was placed 3′ to the *Pet1_210_LA_Cre* construct. *Pet1_210_-Cre* right arm (*Pet1_210_RA*) was generated by means of PCR, using RP23_165_D11 BAC clone as template and the following primers: forward 5′ ATTATTGTCGACAGGTGGTACCAGGGACCAGCC 3′, reverse 5′ ATTATTGTCGACTCGCGCTAGCCGAGTCTGAGC 3′. Upon homologous recombination of the *Pet1_210_-Cre* targeting vector within the RP23_165_D11 BAC, the *Pet1_210_RA* also leads to a deletion of 52 nucleotides downstream the first ATG codon of *Pet1* gene. *Pet1_210_RA* was subcloned into the XhoI restriction site of *Pet1_210_LA_Cre/pSVKeoFRT* plasmid, to generate the *Pet1_210_LA_Cre/pSVKeoFRT/Pet1_210_RA* targeting vector. *Pet1_210_LA_Cre/pSVKeoFRT/Pet1_210_RA* vector and RP23_165_D11 BAC were co-electroporated in *E. coli DY380* cells to obtain the *Pet1_210_-Cre* recombined BAC [39]. After linearization with PI-SceI restriction enzyme, the *Pet1_210_-Cre* recombined BAC was diluted in injection buffer (0.1 mM EDTA; 100 mM NaCl; 10 mM Tris-HCl, pH 7.5; 1x polyamine mix) and microinjected into the male pronucleus of fertilized FVB/N mouse eggs and both presence and copy number of the transgene were assessed by Southern blot analysis. *Pet1_210_-Cre* positive founders were mated to the *ACTB::FLPe* deleter [41] to excise the selection cassette. PCR using primers across the remaining FRT-site (forward: 5′ CGCCTGCTGGAAGATGGCGA 3′; reverse: 5′ CCTTTGGTCCACCGAACTTGC 3′) was performed and amplicon sequencing confirmed that the Flp-mediated recombination occurred correctly. In order to evaluate the integrity of the BAC transgene within the mouse genomic DNA specific primers for the pBACe3.6 backbone were designed as follows: forward 5′ CTAGTAGACTTAATTAAGGATCGAT 3′, reverse 5′ CCGCAAATTTATTAGAGCAATATAG 3′ (5′-end of the PI-SceI linearized transgene, expected amplicon size: 142 bp); forward 5′ CAGGCCTACCCACTAGTCAATT 3′, reverse 5′ TGCTGCTGTTTAGGGATCTGCA 3′ (3′-end of the PI-SceI linearized transgene, expected amplicon size: 254 bp). Transgenic animals were backcrossed to C57BL/6J animals for nine generations to obtain pure C57BL/6J background. Mice were routinely genotyped by PCR in standard conditions using the following primers for *Cre* recombinase: forward 5′ CGCCACGACCAAGTGACAGCA 3′, reverse 5′ CAGGCTAAGTGCCTTCTCTACA 3′.

### Immunohistochemistry

Pregnant females were sacrificed by cervical dislocation, the embryos dissected out of the uterus and fixed o/n in 4% paraformaldeyde (PFA) at 4°C. Adult animals were anaesthetized with Avertin and perfused intracardially with 4% PFA. Brains were dissected out, post-fixed o/n in PFA at 4°C and embedded in either 2.5% agarose or tissue-tek for sectioning with vibratome or cryostat, respectively.

For immunostaining specimens were incubated with primary antibodies o/n at 4°C in PBS containing 5% heat-inactivated lamb serum and 0.5% Triton X-100. Primary antibody dilutions: rabbit anti-5-HT (Sigma) 1∶500; chicken anti-eGFP/eYFP (Abcam) 1∶1000; mouse anti-calbindin-D-28K (Sigma), 1∶200. Fluorescent-conjugated secondary antibody were used as follow: Rhodamine Red-X goat anti-rabbit IgG 1∶500; Alexa Fluor 488 goat anti-chicken IgG 1∶200; Rhodamine Red-X goat anti-mouse IgG 1∶500 (all by Molecular Probes). Cell nuclei were counterstained with DAPI (Sigma), 0.5 µg/ml.

### X-gal chromogenic reaction

X-gal staining was performed on 9.5 dpc, 10.5 dpc, 11.5 dpc, 13.5 dpc *Pet1_210_-Cre*/*ROSA26R* whole embryos and on P1 or P10, P30 and adult *Pet1_210_-Cre*/*ROSA26R* kidney and brains, respectively. Dissected tissues or whole embryos were fixed in 2% formaldehyde solution prepared in PBS for 30 minutes, and subsequently processed for X-gal staining solution containing 5 mM K_4_Fe(CN)_6_, 5 mM K3Fe(CN)_6_, 2 mM MgCl_2_, 0.2% NP40, 0.1% sodium deoxycholate and 1 mg/ml X-gal (Sigma) in PBS for 4–16 h at 30°C. Samples were post-fixed in 4% PFA at 4°C o/n.

β-galactosidase stained specimens were cut at 50 µm with a vibratome or clarified in methyl salicylate pure solution to enhance contrast between X-gal staining and non-stained tissues.

### *In situ* hybridization

*In situ* hybridization was performed as previously described [11]. Briefly, animals were sacrificed by cervical dislocation and fresh brain tissue was dissected out, embedded in Tissue Tek (Sakura), frozen on dry ice and stored at −80°C until used. 14 µm cryostat sections were cut and hybridization was performed according to protocols using either digoxigenin-, fluorescein- or ^35^S-labelled antisense RNA probes. In digoxigenin-labelled *in* *situ* hybridization experiments, NBT/BCIP (Roche) was used as substrate for alkaline phosphatase, while in radioactive *in* *situ* hybridization sections were exposed to Biomax MR X-ray films (Kodak) for two to seven days. For double ISH, sections were hybridized simultaneously with DIG- and fluorescein-labelled probes. A two-step chromogenic reaction using NBT/BCIP and HNPP/Fast Red Fluorescent Detection Set (Roche) was performed to visualize DIG- and fluorescein-labelled riboprobes. Specimens were counterstained with DAPI.

### Image acquisition and data analysis

For brightfield acquisitions, both sections and whole mount samples were observed and photographed with a light microscope or with a MacroFluo microscope equipped with DS-SMc digital cameras (Nikon). Fluorescence images were taken with Eclipse Ti microscopes (Nikon) or with a SP5 confocal microscope (Leica), using 10x and 63x objectives.

For cell counting, double ISH experiments were performed on three distinct C57BL/6J wild-type animals. *Tph2*- and *Pet1*-positive neurons in B9, B8, B7, B5–B6 and B1–B3 *raphe* nuclei were counted using ImageJ software. On average three to four sections depending on the antero-posterior extension of each nucleus were examined. In order to avoid counting cells twice, serial sections 70 µm distance one from another were analysed. For each section two to four 10x images were captured both in brightfield and in TRITC channel to visualize NBT/BCIP, DIG-labelled, or Fast Red, fluorescein-labelled, positive neurons, respectively. Images were converted to 8-bit grayscale and a threshold function was manually applied to remove sub-threshold signal using ImageJ software. For each image, only cells showing labelling clearly above the background level were counted. *Pet1* ^+^/*Tph2* ^+^ and *Pet1* ^+^/*Tph2* ^−^ neurons were then counted per each *raphe* nucleus and the obtained values were expressed as relative percentages.

## Results

### Generation of a *Pet1_210_-Cre* transgenic mouse line driving Cre-mediated recombination in the *raphe* nuclei

In order to minimize positional effect and to guarantee the presence of all *Pet1* regulatory elements, the RP23_165_D11 BAC clone comprising 170 kb upstream and 40 kb downstream the *Pet1* gene locus has been used to drive the expression of Cre recombinase in a transgenic-based approach in the mouse. A homologous recombination strategy in *DY380 E. coli* strain [39] was used to generate the *Pet1_210_-Cre* transgene (Figure S1). After pronuclear injection, Southern Blot analysis on genomic DNA using a probe designed against the *Kana/Neo* DNA sequence allowed the identification of four independent *Pet1_210_-Cre* founders, three of which showed germline transmission, namely founder-female 3 (FF3), founder-female 9 (FF9) and founder-male 3 (FM3). *Pet1_210_-Cre* founders were intercrossed to *ACTB::FLPe* deleter mice [41] to remove the *Kana/Neo* resistance cassette to avoid possible transcriptional interference with the *Pet1* promoter. Genomic DNA was assayed by PCR and sequencing to assess correct Flp-mediated excision of the FRT-flanked *Kana/Neo* cassette (not shown). Eventually, the three *Pet1_210_-Cre* founders were backcrossed to a C57BL/6J background for at least nine generations. The transgenic mice appeared to be morphologically normal, had a normal lifespan and were fertile, thus suggesting no consequences due to the passenger genes (i.e. *Cdk5r2*, *Cryba2*, *Ccdc108*, *Ihh* and *Nhej1*) included in the *Pet1_210_-Cre* BAC construct (Figure S1).

In order to characterize the newly generated transgenic lines, we first tested the Cre somatic recombination efficiency by intercrossing *Pet1_210_-Cre* transgenic animals obtained from the 3 distinct founders to the *ROSA26R* conditional reporter line, in which β-galactosidase is constitutively expressed upon Cre-mediated recombination [42].

**Figure 1:**
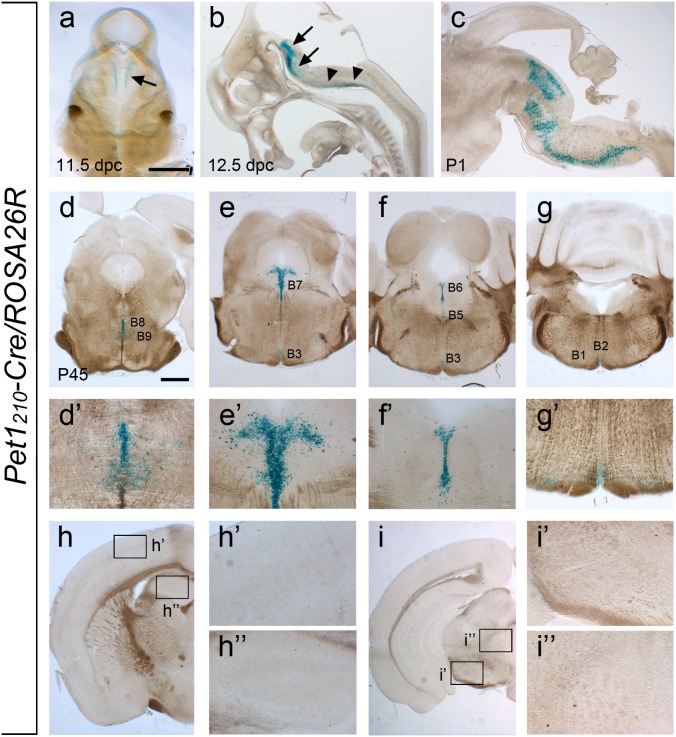
*Pet1_210_-Cre* mouse line drives Cre recombinase activity in the serotonergic system. (**a**) Dorsal view of a cleared X-gal stained *Pet1_210_-Cre/ROSA26R* embryo at 11.5 dpc showing that Cre-mediated somatic recombination has occurred in the rostral *raphe* (arrow). (**b**) Sagittal section of 12.5 dpc *Pet1_210_-Cre/ROSA26R* embryo highlighting the presence of the reporter in both the rostral (arrows) and caudal (arrowheads) clusters of developing serotonergic neurons. (**c**) In P1 *Pet1_210_-Cre/ROSA26R* brains X-gal staining highlights serotonergic neurons migrated towards their terminal locations within the rhombencephalon. (**d–g, d’–g’**) Representative coronal sections throughout the antero-posterior extent of the *raphe* of an adult (P45) *Pet1_210_-Cre/ROSA26R* brain showing Cre-mediated recombination specifically occurred in all serotonergic nuclei, namely B8–B9 (**d, d’**), B7 (**e, e’**), B5–B6 (**f, f’**) and B1–B3 (**g, g’**). (**h-h’’**, **i-i’’**) no β-galactosidase staining is detectable in anterior brain regions such as cortex (**h-h’**), hippocampus (**h, h’’**), substantia nigra (**i-i’**) and thalamus (**i, i’’**). Scale bar: 1 mm (**a**, **d–i**), 900 µm (**c**), 600 µm (**b**), 300 µm (**d’**-**i’**, **h’’**, **i’’**).

It is reported that in the mouse hindbrain *Pet1* expression starts around 10.5 dpc in the rostral serotonergic domain, and about one day later in the caudal *raphe* nuclei [31], [32]. X-gal staining analyses revealed that Cre-mediated recombination had occurred already in the hindbrain of 11.5 dpc *Pet1_210_-Cre/ROSA26R* mouse embryos showing two longitudinal dark blue stripes lateral to the floorplate defining the rostral serotonergic domain. Conversely, β-galactosidase staining in the medullary domain was barely detectable at this stage, reflecting the rostro-caudal temporal order in the generation of *raphe* serotonergic neurons (Figure 1a). At 12.5 dpc the analysis on sagittal sections and hindbrain flat-mount preparation from the three distinct founders showed that the transgene was expressed both in the anterior r1-r3-derived and in the posterior r5-r7-derived hindbrain regions, with the exception of the r4-derived territory, thus mirroring the endogenous expression of the *Pet1* gene (Figure 1b and Figure S2 a–c). At P1, β-galactosidase-expressing neurons have migrated from their original position in the ventral region of the hindbrain and reached their final location in the brainstem, defining dorsal, medial and medullary clusters of serotonergic neurons (Figure 1c), in line with the morphogenetic movements of the developing serotonergic system occurred at this stage [43]. β-galactosidase staining was confirmed in all serotonergic B1–B9 *raphe* nuclei of adult *Pet1_210_-Cre/ROSA26R* transgenic mice (Figure 1d–g, d’-g’), but not in ectopic districts as assessed by a detailed analysis on coronal sections throughout *Pet1_210_-Cre/ROSA26R* mouse brains (Figure 1h–i, h’-i’, h’’-I’’). Finally, we compared β-galactosidase staining pattern to *Pet1* mRNA distribution at both foetal (i.e. 12.5 dpc and 15.5 dpc) and postnatal stages (i.e. P10 and P30). Results showed that the transgene expression nicely correlates to *Pet1* endogenous expression (Figure S3), and to that of the specific marker of terminally differentiated 5-HT neurons such as *Tph2*. Thus, in the *Pet1_210_-Cre* transgenic mouse line *Cre recombinase* expression likely mirrors *Pet1* spatio-temporal localization in the *raphe* nuclei during both foetal development and post-natal life. As the three founders showed similar Cre activity, we selected the FF9-derived mice, which showed the strongest β-galactosidase signal, to perform the detailed analysis described below. For this founder further genomic analysis was performed in order to assess the integrity of the BAC transgene and the transgene copy number integrated into the genome. Evidence that the BAC transgene was intact within the chromosomal DNA of *Pet1_210_-Cre* mice was deduced by the amplification of the 142 bp and 254 bp fragments corresponding to the 5′- and 3′-end, respectively, of the PI-SceI linearized BAC backbone (Figure S1 c). Furthermore, Southern blot analysis showed that the BAC transgene was integrated as a single copy into the genome of FF9-derived mice (Figure S1 d).

### Identification of a non-serotonergic *Pet1* ^+^ cell population in the *raphe*

**Figure 2:**
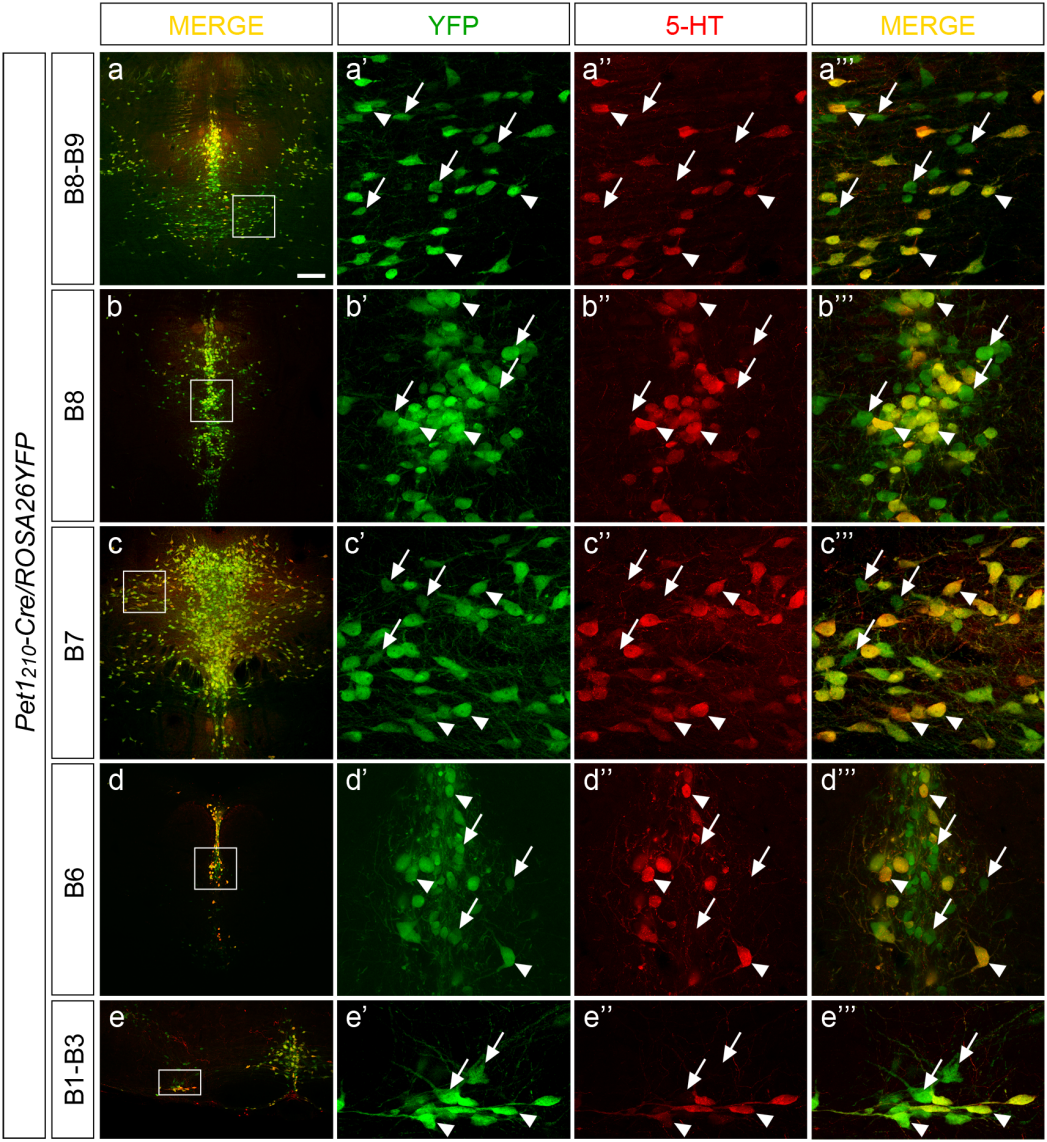
*Pet1_210_-Cre* transgenic mouse line promotes somatic recombination in a non-serotonergic cell population within the *raphe* nuclei. Representative low (**a**, **b**, **c**, **d**, **e**) and high (**a’-a’’’**, **b’-b’’’**, **c’-c’’’**, **d’-d’’’**, **e’-e’’’**) magnification confocal images of coronal sections of adult *Pet1_210_-Cre/ROSA26YFP* brains, showing the distribution of YFP (green) and 5-HT (red) immunoreactivity within B8–B9 (**a-a’’’**), B8 (**b-b’’’**), B7 (**c-c’’’**), B6 (**d-d’’’**), and B1–B3 (**e-e’’’**) serotonergic nuclei. Boxes highlight the region of each *raphe* nucleus shown at higher magnification. Cells immunoreactive for both YFP and 5-HT (YFP^+^/5-HT^+^, arrowheads) or exclusively YFP but not 5-HT (YFP^+^/5-HT^−^, arrows) were detected along the antero-posterior extent of the *raphe* highlighting variable representativeness among the different nuclei. Scale bar: 200 µm (**a–e**), 30 µm (**a’-a’’’**, **b’-b’’’**, **c’-c’’’**, **d’-d’’’**, **e’-e’’’**).

We then intercrossed the *Pet1_210_-Cre* mice with the *ROSA26YFP* conditional reporter line [44], in which the *YFP* reporter gene is constitutively activated upon Cre expression, in order to assess the Cre-mediated recombination activity at a cellular level. Combined double immunohistochemistry experiments were performed using specific antibodies against YFP and 5-HT on sections from *Pet1_210_-Cre*/*ROSA26YFP* double transgenic mouse brains (Figure 2 a–e).

Results highlighted a discrepancy between YFP and 5-HT immunoreactivity within the *raphe* nuclei along the rostro-caudal extent of the hindbrain. In particular, while virtually all 5-HT-immunoreactive cells resulted positive for YFP (YFP^+^/5-HT^+^), several YFP-immunoreactive cells resulted to be devoid of serotonin (YFP^+^/5-HT^−^, Figure 2 a’-a’’’, b’-b’’’, c’-c’’’, d’-d’’’, e’-e’’’). In particular, the fraction of YFP^+^/5-HT^−^ vs YFP^+^/5-HT^+^ cells displayed a rostral-to-caudal decreasing ratio, being sizable in the rostral median *raphe* (MR) B8-B9 nuclei (Figure 2 a-a’’’, b’-b’’’), while in dorsal *raphe* (DR) B7 (Figure 2 c-c’’’), B6 (Figure 2 d-d’’’) and caudal B1–B3 nuclei (Figure 2 e-e’’’) only few YFP^+^/5-HT^−^ were present.

**Figure 3:**
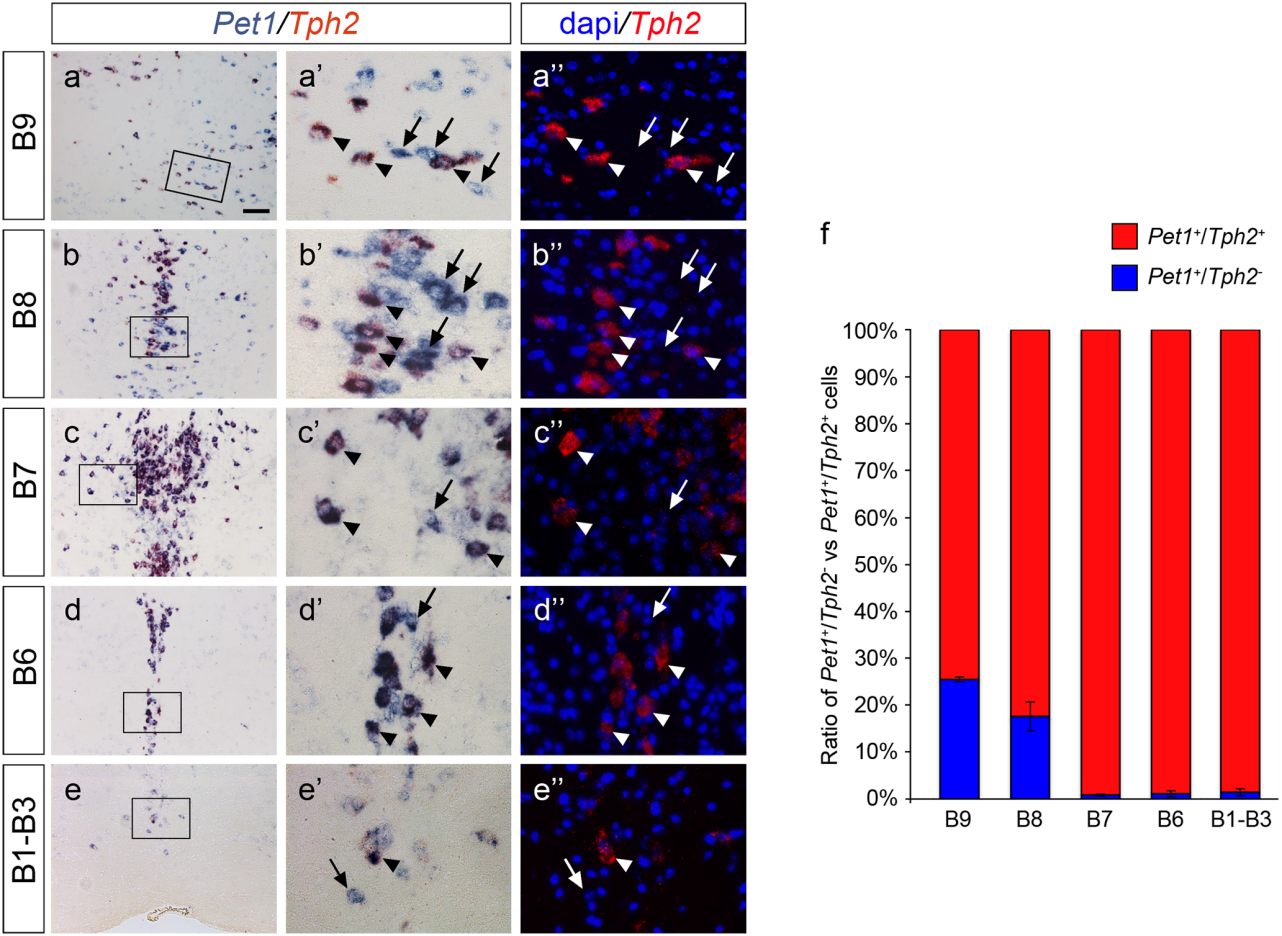
*Pet1* gene is expressed in a non-serotonergic *raphe* cell population in adult mice. (**a–e’’**) Low (**a**, **b**, **c**, **d**, **e**) and high (**a’-a’’**, **b’-b’’**, **c’-c’’**, **d’-d’’**, **e’-e’’**) magnification images of double ISH performed on coronal sections obtained from adult wild-type mice at the level of B9 (**a-a’’**), B8 (**b-b’’**), B7 (**c-c’’**), B6 (**d-d’’**), and B1–B3 (**e-e’’**) *raphe* nuclei. In each picture, *Pet1* expression is highlighted by a dark blue staining (**a-a’**, **b-b’**, **c-c’**, **d-d’**, **e-e’**), while *Tph2* gene expression is visualized as a red precipitate (**a-a’**, **b-b’**, **c-c’**, **d-d’**, **e-e’**), or as a red fluorescence (**a’’**, **b’’**, **c’’**, **d’’**, **e’’**). Boxed areas indicate the regions shown in higher magnification images. In all the *raphe* nuclei two distinct populations of neurons expressing either both *Pet1* and *Tph2* (arrowheads), or only *Pet1* (arrows) are present. (**f**) Ratio of *Pet1^+^*/*Tph2^+^* vs *Pet1^+^*/*Tph2^−^* neuronal population in distinct *raphe* nuclei of adult wild-type mice reported as percentage. Histogram shows that *Pet1*-positive non-serotonergic neurons are significantly represented in rostral B8 and B9 as compared to more posterior nuclei. Data are presented as mean ± SEM. Scale bar: 100 µm (a–e), 25 µm (**a’-a’’**, **b’-b’’**, **c’-c’’**, **d’-d’’**, **e’-e’’**).

**Figure 4:**
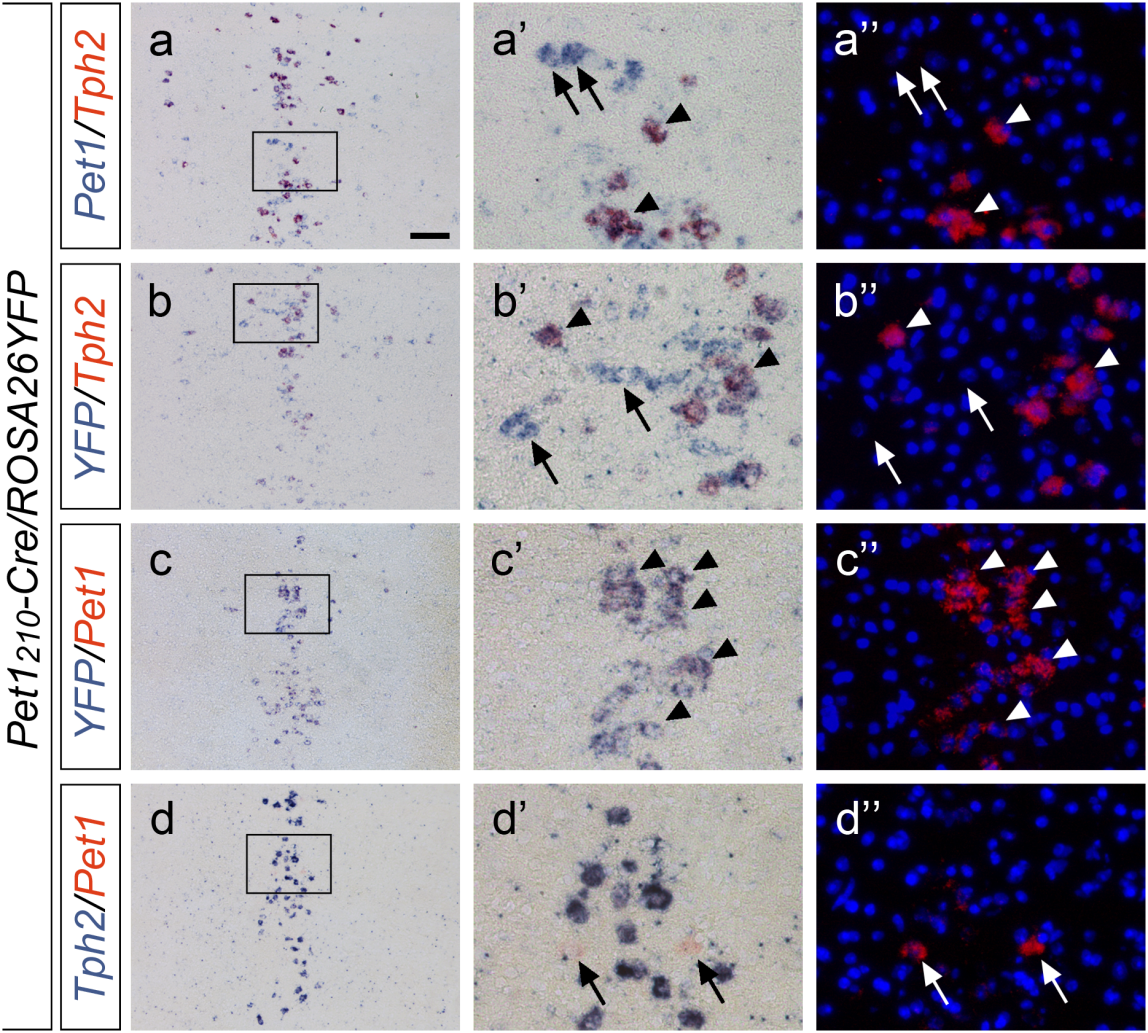
*Pet1_210_-Cre* transgene specifically identify *Pet1*-expressing, non-serotonergic *raphe* neurons. Double ISH performed on serial coronal sections of adult *Pet1_210_-Cre/ROSA26YFP* mouse brains at the level of B8 *raphe* nucleus using combination of *Pet1*/*Tph2* (**a-a’’**), *YFP*/*Tph2* (**b-b’’**), *YFP*/*Pet1* (**c-c’’**) and *Tph2*/*Pet1* (**d-d’’**) riboprobes. In each combination the former probe is highlighted using the NBT/BCIP substrate, while the latter using the Fast Red chromogen. Boxed areas are shown at higher magnification in brightfield (**a’**, **b’**, **c’**, **d’**) or fluorescence (**a’’**, **b’’**, **c’’**, **d’’**). While all *Pet1*-positive cells also express *YFP* (arrowheads in **c’-c’’**), the presence of a *Pet1*-positive, non-serotonergic cell population was confirmed with all the other probe combinations (arrows in **a’-a’’**, **b’-b’’**, **d’-d’’**). Scale bar: 100 µm (**a**–**d**), 25 µm (**a’**-**a’’**, **b’**-**b’’**, **c’**-**c’’d’**-**d’’**).

Thus, our results raised the question whether the unexpected presence of YFP^+^/5-HT^−^ cells in the adult mouse brain could be due to an ectopic activity of the Cre recombinase or to a specific, novel expression domain of *Pet1* gene in a non-serotonergic cell subpopulation. To answer this question, we performed combined double *in* *situ* hybridization experiments on adult wild-type mice using specific riboprobes for both *Pet1* and *Tph2*, with the latter being expressed selectively in all terminally differentiated serotonergic neurons within the *raphe* nuclei (Figure 3 a–e). Consistently with immunohistochemistry data, few neurons expressing *Pet1* but devoid of *Tph2* were detected in dorsal and caudal *raphe* B6 (Figure 3 d-d’’), B7 (Figure 3 c-c’’) and B1–B3 (Figure 3 e-e’’) nuclei whereas in B9 (Figure 3 a-a’’) and B8 (Figure 3 b-b’’*) raphe* nuclei a substantial number of non-serotonergic neurons resulted positive for *Pet1* expression. In order to quantify this observation, both *Pet1^+^/Tph2^+^* and *Pet1^+^/Tph2^−^* neurons were counted in B9, B8, B7 and B6 rostral *raphe* nuclei and in the B1–B3 caudal cluster. Analysis showed that in B1–B3 group, in B6 and in B7 *raphe* nuclei the percentage of *Pet1^+^/Tph2^−^* neurons was 1.5%, 1.1% and 0.8%, respectively, while in B8 and in B9 nuclei it reached 17.6% and 25.5%, respectively (Figure 3f). Further, to address at the cellular level whether in our *Pet1_210_-Cre* transgenic mouse line the Cre recombinase promoted somatic recombination mirroring the *Pet1* expression pattern, we used two distinct riboprobe combinations (i.e. *Pet1* and *YFP*, or *Tph2* and *YFP*) to perform double ISH on serial coronal sections of adult *Pet1_210_-Cre*/*ROSA26YFP*. Analyses of results confirmed that the reporter *YFP* is present in all *Pet1* ^+^ neurons as highlighted by co-expression of *Pet1* and *YFP* (Figure 4 c-c’’), and demonstrated that the *Pet1* promoter is also active in non-serotonergic neurons as shown by the partially overlapping expression of *Tph2* and *YFP* (Figure 4 b-b’’), in line with the presence of a *Pet1* ^+^ */Tph2* ^−^ neuronal population in the *raphe* system (Figure 4 a-a’’). Moreover, as NBT/BCIP deep purple chromogenic precipitate may quench the fluorescence generated by the HNPP/Fast Red substrate, masking the *Tph2* signal in *Pet1* positive neurons, we repeated double ISH on coronal sections at the level of B8 *raphe* nucleus swapping the detection methods for *Pet1* (HNPP/Fast Red) and *Tph2* (NBT/BCIP) riboprobes as an additional control. We intentionally let the staining with NBT/BCIP substrate proceed until saturation was reached, so that *Tph2* expressing neurons showed a very dark blue signal likely masking any underlying fluorescence. In spite of that, *Pet1*-only positive neurons identified by alkaline-phosphatase activity using HNPP/Fast Red substrate were still clearly visible (Figure 4 d-d’’). Together, these data indicate that *Pet1_210_-Cre* mouse line promotes *Cre recombinase* expression in all serotonergic neurons and identifies for the first time a novel, non-serotonergic *raphe* neuronal population expressing *Pet1*.

### *Pet1* gene is expressed in developing pancreas and kidney

**Figure 5:**
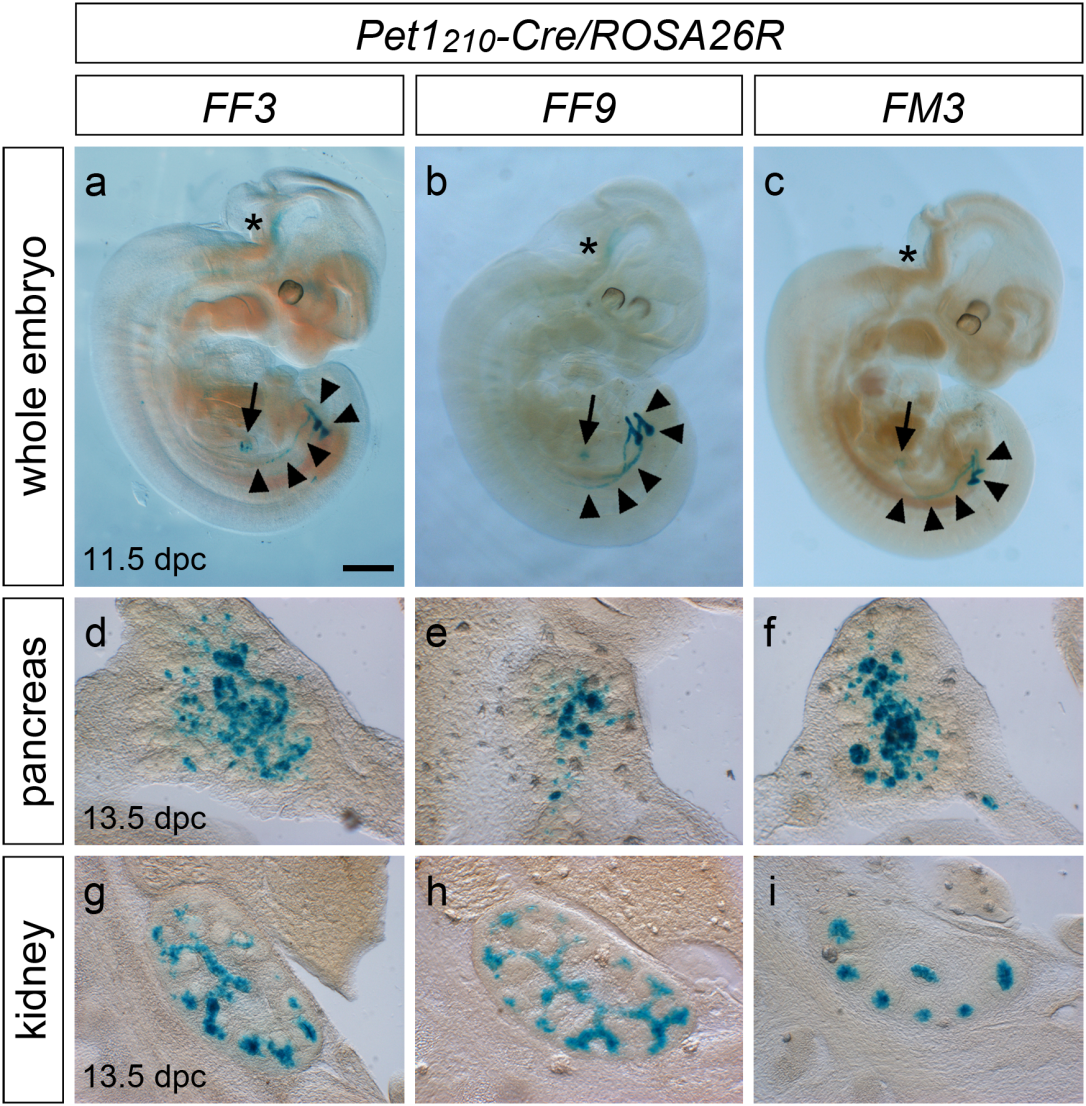
*Pet1* drives Cre-mediated recombination in the developing pancreas and kidney. X-gal staining performed on *Pet1_210_-Cre/ROSA26R* whole mount 11.5 dpc embryos (**a–c**), and on sagittal sections at the level of the developing pancreas (**d–f**) and kidney (**g–i**) of 13.5 dpc embryos obtained from FF3 (**a**, **d**, **g**), FF9 (**b**, **e**, **h**) and FM3 (**c**, **f**, **i**) founder mouse lines. At 11.5 dpc a clear expression of the reporter is present at comparable levels in the three founder lines in both the developing pancreas (arrows) and kidneys (arrowheads), while it is still barely detectable in the hindbrain (asterisks). At 13.5 dpc X-gal staining highlights cells in the developing pancreas (**d–f**) and in the branching ureteric bud of the kidney (**g–i**) where Cre-mediated somatic recombination has occurred. Scale bar: 600 µm (**a**–**c**), 150 µm (**g**–**i**), 100 µm (**d**–**f**).

Analysis of the spatio-temporal domain of the Cre recombinase activity in the *Pet1_210_-Cre/ROSA26R* mouse embryos from all the three founders at both 11.5 dpc and 13.5 dpc revealed the presence of β-galactosidase staining in additional potential *Pet1* expression domains outside the CNS unprecedentedly described (Figure 5). At 11.5 dpc Cre-mediated recombination was identified in the anlage of the pancreas and in the ureteric bud of the developing kidney, which at this stage forms a small branch at the level of the hindlimbs at the terminal extremity of tubular structures on each side of the caudal abdominal region (Figure 5 a–c). At 13.5 dpc, X-gal staining performed on parasagittal sections of *Pet1_210_-Cre/ROSA26R* embryos corroborated the presence of Cre recombinase activity in the developing pancreas and in the branching ureteric bud of the kidney (Figure 5 d–i). Additionally, X-gal staining performed on whole organs or sections obtained from *Pet1_210_-Cre/ROSA26R* pups at birth or adult animals, showed that while both pancreas and kidneys confirmed the presence of X-gal staining, no reporter expression was evident in all the other *Pet1_210_-Cre/ROSA26R* organs analysed (Figure S4).

The presence of β-galactosidase activity in both pancreas and kidneys of mice derived from all the *Pet1_210_-Cre* founders prompted us to hypothesize that the expression of the conditional reporter was due to specific, previously unreported *Pet1* expression domains, rather than an ectopic activation of Cre recombinase. To address this hypothesis we performed ISH experiments on pancreas and kidneys from 15.5 dpc *Pet1_210_-Cre/ROSA26YFP* embryos, using specific riboprobes for *Pet1* and *YFP*. Results indicated that *YFP* and *Pet1* are expressed in the same pancreatic domains, nicely correlating with the pancreatic islet cell marker *Nkx2.2* (Figure S5 a–c). Moreover, despite in the hindbrain the expression of *Pet1* normally preludes to the acquisition of a serotonergic phenotype, we could not detect either *Tph2* or *Tph1* expression in the developing pancreas (Figure S5 d–e).

**Figure 6:**
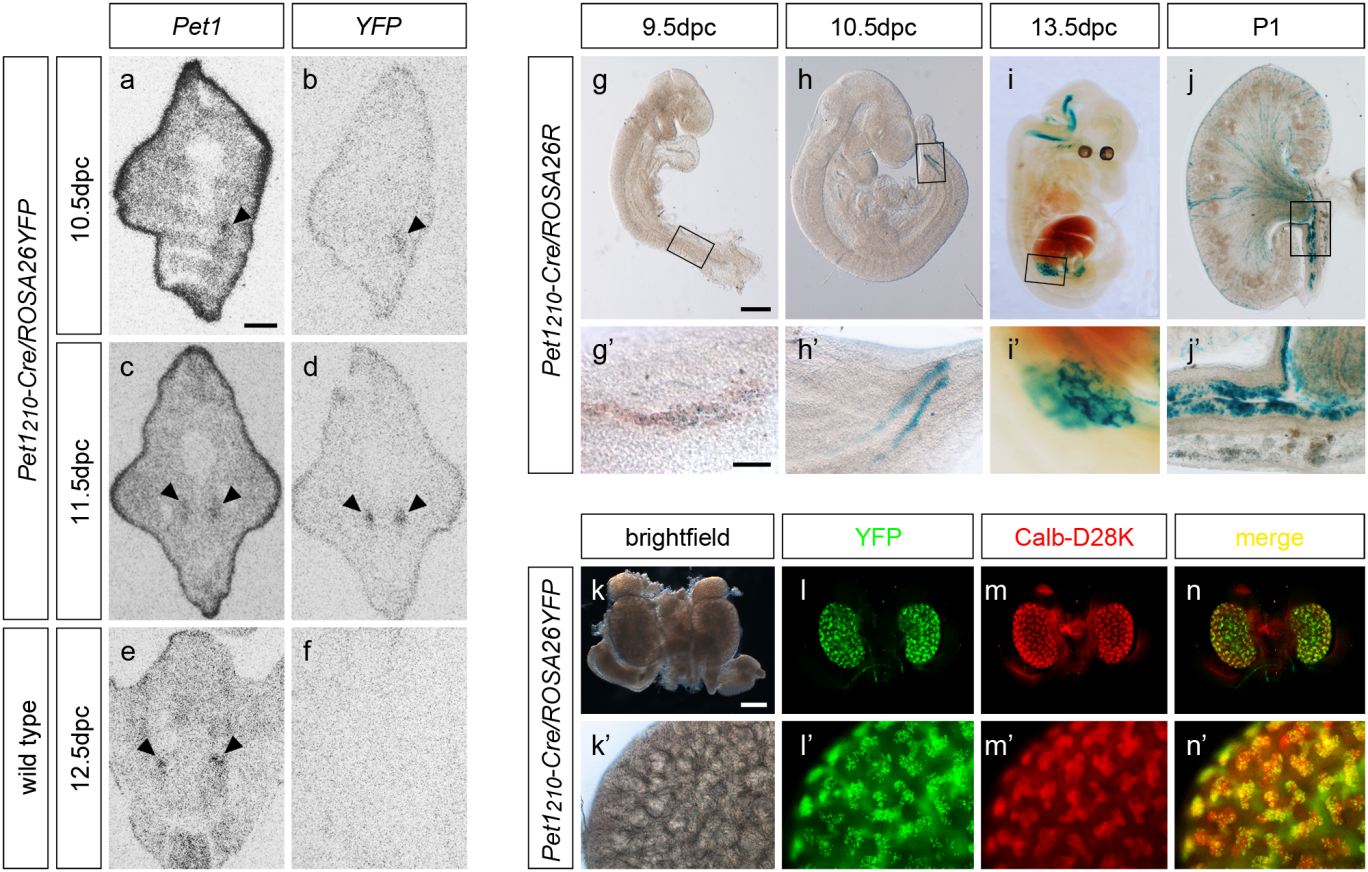
*Pet1* is expressed in the ureteric bud-derived tissues during kidney development. (**a–f**) Radioactive ISH performed on coronal section of 10.5 dpc (**a–b**), 11.5 dpc (**c–d**) and 12.5 dpc (**e–f**) *Pet1_210_-Cre/ROSA26YFP* (**a–d**) and wild-type (**e–f**) embryos. Results show presence of *Pet1* mRNA expression in the nephric duct (arrowheads) already at 10.5 dpc (**a**), mirroring YFP reporter expression (**b**). At 11.5 dpc both *Pet1* and *YFP* expression is highlighted in the two forming ureteric buds (**c–d**, arrowheads), while *Pet1* but not the reporter expression is detectable in 12.5 dpc wild-type embryos (**e–f**), confirming the specificity of *Pet1* expression in the developing kidney. (**g–j’**) X-gal staining performed on whole-mount 9.5 dpc (**g**, **g’**), 10.5 dpc (**h**, **h’**), 13.5 dpc (**i**, **i’**) *Pet1_210_-Cre/ROSA26R* mouse embryos and on coronal sections of a P1 *Pet1_210_-Cre/ROSA26YFP* mouse kidney (**j**, **j’**). Staining performed at different stages of development traces *Pet1*-expressing cell lineage during ureteric bud formation as highlighted in boxed regions shown in the higher magnification images (**g’**, **h’**, **i’**). Cre-mediated recombination has already occurred at 9.5 dpc in some scattered cells in the caudal nephric duct (**g’**). X-gal stained cells become more numerous at 10.5 dpc (**h’**) and depict the ureteric bud branching at 13.5 dpc (**i’**). At P1, X-gal staining highlights that Cre expressing cells have contributed to the formation of the collecting duct system (**j**) and ureter (**j’**). (**k–n’**) Brightfield (**k**, **k’**) and fluorescence images of whole-mount 13.5 dpc *Pet1_210_-Cre/ROSA26YFP* kidneys immunostained for YFP (**l**, **l’**) and calbindin-D28K (**m**, **m’**). Merge images (**n**, **n’**) show colocalization of Cre recombinase activity and the specific ureteric bud-derivative marker calb-D28K during kidney development. Scale bar: 1.2 mm (**i**), 600 µm (**h**), 500 µm (**e**, **f**, **k**–**n**), 400 µm (**a**–**d**, **g, j**), 200 µm (**h’**–**i’**), 100 µm (**j’, k’**–**n’**), 80 µm (**g’**).

To assess the presence of *Pet1* mRNA in the kidney, due to its low expression level, we performed radioactive ISH on coronal sections of 10.5 dpc, 11.5 dpc and 12.5 dpc from *Pet1_210_-Cre/ROSA26YFP* embryos, using specific ^35^S-labeled riboprobes against *YFP* and *Pet1* (Figure 6 a–f). Analysis of autoradiograms showed detectable *Pet1* expression in the developing kidney, which correlated with *YFP* expression in double *Pet1_210_-Cre/ROSA26YFP* transgenic embryos at 10.5 dpc (Figure 6 a–b). At 11.5 dpc radioactive ISH showed that *Pet1* is expressed in the nephric ducts at the level of the hindlimbs, with a similar pattern of *YFP* mRNA distribution (Figure 6 c–d). Radioactive ISH performed as a control on 12.5 dpc wild-type embryos, showed that no *YFP* expression was detectable, while *Pet1* expression was still present in the kidney at this stage (Figure 6 e–f).

In order to fate map the *Pet1* expressing cells in the developing kidneys, we performed β-galactosidase staining on whole mount *Pet1_210_-Cre/ROSA26R* specimens at different developmental stages relevant in kidney development, from 9.5 dpc to P1 (Figure 6 g–j), and in adult (Figure S4). The expression of the reporter became detectable as early as 9.5 dpc in scattered cells along the developing nephric duct (Figure 6 g-g’). At 10.5 dpc X-gal staining was present in the nephric duct showing an increasing intensity toward its caudal end (Figure 6 h-h’). At 13.5 dpc β-galactosidase staining nicely highlighted that ureteric bud underwent already through different rounds of branching forming a tree-like structure (Figure 6 i-i’). Finally, in P1 mice X-gal staining was present in cells belonging to the collecting duct epithelium (Figure 6 j-j’). Importantly, no X-gal staining was observed within the metanephric mesenchyme, thus suggesting that in *Pet1_210_-Cre* transgenic mouse line Cre-mediated recombination occurred specifically within the ureteric bud precursors. No Cre recombinase activity was detected in the adrenal gland of *Pet1_210_-Cre/ROSA26R* mice (Figure S4 c, i-i’) as expected in agreement with Fyodorov and collaborators (1998) [45].

In order to confirm that *Pet1* expression is localized within the ureteric bud derivatives, we performed immunohistochemistry experiments on *Pet1_210_-Cre/ROSA26YFP* whole mount kidneys at 13.5 dpc using antibodies against YFP and the specific marker for ureteric bud calbindin-D28K [46]. Results showed that cells expressing YFP were also immunoreactive for calbindin-D28K, which stains both tips and stalks of ureteric bud branches (Figure 6 k–n, k’-n’). In contrast, not all calbindin-D-28K^+^ cells expressed YFP, indicating that Cre-mediated recombination in the kidney occurred in a subset of calbindin-D28K expressing cells.

Thus, the characterization of the *Pet1_210_-Cre* transgenic line allowed the identification of novel expression domains outside the CNS where Cre activity faithfully recapitulates *Pet1* expression.

## Discussion

In the present study we reported the generation and the comprehensive characterization of the *Pet1_210_-Cre* transgenic mouse line, in which a BAC-derived 210 kb genomic fragment of *Pet1* gene locus was used to drive *Cre recombinase* expression. To date, it represents the largest *Pet1* genomic region used to drive *Pet1*-like transgene expression. We observed that in *Pet1_210_-Cre* transgenic mouse line the 210 kb region driving *Cre recombinase* expression was able to recapitulate the timing of the endogenous *Pet1* expression during serotonergic system development, as well as the spatial distribution within B1–B9 *raphe* nuclei. This result, together with the absence of reporter expression in extra-*raphe* domains in the brain, indicated a reliable, *Pet1-*driven, spatial and temporal control of *Cre* expression in the *Pet1_210_-Cre* mouse line. These results are in line with the peculiarities of BAC-based transgenesis, that limits positional effects and guarantees the presence of long-range acting regulatory elements as well, likely promoting transgene expression in an endogenous-like manner independently of the integration site [34], [36]–[38], [47]. Furthermore, despite the expression of *Pet1* in serotonergic neurons observed in our *Pet1_210_-Cre* transgenic mice is consistent with data previously obtained with other *Pet1*-based transgenic mouse lines [16], [22], [48], [49], in the present study we demonstrated that *Pet1* expression in the *raphe* is present in a wider domain than previously described, as assessed by double IHC characterization of *Pet1_210_-Cre* transgenic line and by ISH experiments on wild-type animals with different combination of *Pet1* and *Tph2* probe staining. Interestingly, we observed that within the *raphe* system the non-serotonergic *Pet1*-positive neurons are unevenly rather than homogeneously distributed along the *raphe*, with a substantial number in B8 and B9 nuclei, as compared to DR and caudal cluster B1–B3, where only few *Pet1* ^+^/*Tph2* ^−^ cells were present.

It has been well established that the majority of neurons populating the *raphe* are not serotonergic, even though the term *raphe* is often used as a synonym of serotonergic [50]–[52]. Both dorsal *raphe* and median *raphe* nuclei, which together provide the main serotonergic innervation to the brain, are also composed by a heterogeneous population of neurons expressing different transmitter substances, such as glutamate [53], GABA [54], or peptides (e.g. corticotropin-releasing factor and substance P) [29]. The composition and the distribution of non-serotonergic neurons, as well as serotonergic neurons, show distinct pattern in DR and MR nuclei, often reflecting different target brain regions or subregions of the same brain structure such as amygdala, hippocampus and medial septum among the others [28], [50], [55]–[57]. Retrograde tracing studies have demonstrated that a large population of non-serotonergic neurons of the MR expresses the vesicular glutamate transporter VGLUT3, while in DR serotonin and VGLUT3 are often overlapping [57], [58]. The evidence that other transmitter substances may be co-released with serotonin hampers the study of non-serotonergic populations of the *raphe* and, therefore, the possibility to better characterize the *Pet1* ^+^/*Tph2* ^−^ neuronal population. Although beyond the intent of the present study, multiple labelling studies would be of great interest in future research in order to identify the molecular and neurotransmission characteristics of *Pet1* ^+^/*Tph2* ^−^ cells. In this regard, intersectional and subtractive strategies have provided powerful tools to map cell subtypes with great precision [22], [59], [60]. Jensen and collaborators have shown that serotonergic neurons can be genetically defined on the basis of their rhombomeric origin more than their final localization in adult serotonergic nuclei. In particular, it has been reported that while DR (B7, B6, B4) neurons derive uniquely from r1, median nuclei (B9, B8, B4) derive from r1–r3 precursors [22]. Thus, in line with this study, it can be hypothesized that the *Pet1*-positive non-serotonergic neurons present in *raphe* nuclei likely arise from a heterogeneous population of precursors deriving from r1 to r3. The combination of suitable transgenic and conditional reporter mouse lines in an intersectional approach may represent a valuable tool to dissect the developmental origin and the nature of *Pet1*-positive, non 5-HT neurons. The possibility to shed light on the molecular identity of *Pet1^+^/Tph2^−^* neurons will potentially impact on those studies in which the *Pet1* regulatory region has been used to drive *Cre recombinase* expression, in order to specifically target serotonergic neurons. Indeed, in light of our results, the non-serotonergic expression might have contributed to the observed phenotypes resulting from *Pet1*-driven Cre-mediated somatic recombination in conditional knockouts.

The use of *Pet1_210_-Cre* transgenic mouse line allowed the tracing of *Pet1* expressing cell progeny in the developing pancreas. Indeed, our data are consistent with the report by Ohta and collaborators, who have shown, using the *ePet-Cre* transgenic mouse line [61], that *Pet1* is expressed in both developing and adult pancreas, peaking at E14.5 and co-localizing with endocrine-specific markers such as glucagon and insulin or specific transcription factors as *Nkx2.2*. In the same study, the analysis of *Pet1* mutant animals showed a reduction of the expression of insulin genes, resulting in glucose clearance and insulin secretion defects. Interestingly, *Pet1* does not directly promote *Tph2* expression in the pancreas, suggesting that the *Pet1*-mediated regulation of either pancreatic β-cell or serotonergic neuron development proceeds via distinct genetic cascades [61]. More importantly, to our knowledge the present work provides the first evidence of *Pet1* expression in kidney. Lack of previous reports showing *Pet1* expression in the ureteric bud derivatives may be due to a sub-threshold expression level and/or to a transient expression within this structure. In this regard, an approach based on Cre/loxP system that induces a sustained reporter expression upon somatic recombination can reveal both sub-detectable and transient expression domains, that otherwise would be difficult to label with canonical immunohistochemistry- or *in* *situ* hybridization-based methods.

Our findings on the spatio-temporal expression domain of *Pet1* go beyond the results obtained using the previously generated *ePet1-Cre* transgenic mouse line, in which presence of *Pet1* in the kidney was excluded [62]. This discrepancy might be due to a number of factors, with the most likely being that the regulatory elements necessary to drive a *Pet1*-like expression in the kidney are likely included in the 210 kb *Pet1* entire regulatory region of the *Pet1_210_-Cre* transgene, and not present in the 40 kb region used to generate the *ePet1-Cre* transgene [62]. In good agreement with our observation that *Pet1* is expressed in mammalian kidney, microarray analysis performed on mouse and rat transcriptomes indicated that *Pet1* transcript was enriched in the ureteric bud as compared to other cell types in the developing kidney such as the metanephric mesenchyme [63]. Nevertheless, although *Pet1* expression has been shown in the adrenal gland by Fyodorov and collaborators [45], Cre recombinase activity in this district was not detected either by Scott and collaborators [16], or from our analysis, using *ePet-Cre* and *ePet-YFP*, or *Pet1_210_-Cre* transgenic mouse lines, respectively. The presence of an intact recombined BAC in *Pet1_210_-Cre* mice suggests that the specific enhancer driving expression of *Pet1* in the adrenal gland may be a long-range acting regulatory element, which is not likely present in the 210 kb genomic region contained in the RP23_165_D11 BAC clone.

Interestingly, as already observed for pancreas development [61], also in the kidney *Pet1* is co-expressed with other transcription factors shared with the serotonergic differentiation pathway. It has been reported that *LMX1B* mutation causes nephropathies and, often, renal failure in human patients affected by Nail-Patella syndrome [64], [65]. Mouse *Lmx1b* is expressed in podocytes of the kidney, and its genetic ablation results in kidney defects resembling those observed in Nail-Patella syndrome patients [66], [67]. On the other hand, the functional role of *Pet1* in the kidney has not been yet characterized. It is known that chromosomal translocations in which *Ewing’s sarcoma* (*EWS*) gene is fused to a variety of transcription factors, including human *FEV*, can lead to the onset of different subsets of Ewing tumours [68], [69], with some of them occasionally occurring in the kidney [70].

On the whole, the use of *Pet1_210_-Cre* transgenic mouse line allowed the identification of novel *Pet1* expression domains both in the hindbrain and in the developing kidney. As the expression of the reporter is permanently induced following recombination, this line will allow long-term analysis of *Pet1*-expressing cell types, both in serotonergic neurons as well as in non-serotonergic cell populations of the *raphe*. Moreover, this mouse line constitutes a valuable model for studying molecular mechanisms of renal and pancreatic development and function by induction or silencing of specific genes in epithelial ureteric bud-derivatives and islet cells, respectively. Finally, given the expression of *Pet1* in extra-serotonergic neurons within the *raphe* system, when using *Pet1*-driving Cre transgenic mouse lines in conditional gene targeting approaches or fate mapping studies aimed to target serotonergic cells, it should be taken into great consideration that the phenotype observed might arise from a combined, rather than serotonergic-specific, somatic recombination.

## Supporting Information

Figure S1 ***Pet1_210_-Cre*** **allele generation.** Diagram showing the wild-type *Pet1* genomic locus contained within the RP23_165D11 BAC clone (**a**), the targeting vector for the homologous recombination in E. coli *DY380* cells and the resulting *Pet1_210_-Cre* transgenic allele before and following Flp-mediated recombination. (**b**) Southern Blot analysis performed on genomic DNA obtained from *Pet1_210_-Cre* founders after BamHI digestion and hybridization with a probe against *Kana/Neo* cassette. Among the founders analysed, 4 presented the expected band at 2.6 kb. Founder-male 3 (FM3, lane 1), founder-female 3 (FF3, lane 7) and founder-female 9 (FF9, lane 10) showed germline transmission. (**c**) A 142 bp fragment at the 5′-end and a 254 bp fragment at the 3′-end of the transgene were amplified from the *Pet1_210_-Cre* FF9 founder genomic DNA and from the pBACe3.6 backbone assessing the integrity of the BAC transgene within mouse genomic DNA. (**d**) Southern Blot analysis performed on genomic DNA obtained from *Pet1_210_-Cre* FF9 founder after NotI digestion and hybridization with a probe capable to discriminate wt *Pet1 locus* vs *Pet1_210_-Cre* transgene. A clear lower hybridization intensity of the *Pet1_210_-Cre* transgene (4.2 kb) as compared to the *Pet1* wt allele (3.1 kb) confirms the presence of a single copy of the transgene. L: ladder; wt: wild-type genomic DNA; tg+: *Pet1_210_-Cre* FF9 derived genomic DNA; BAC: RP23_165D11 BAC clone.
(TIF)

Figure S2 ***Pet1*** **drives Cre-mediated recombination in the serotonergic domains of the three** ***Pet1_210_-Cre*** **founders.** Flat mount preparations of 12.5 dpc hindbrains obtained from *Pet1_210_-Cre/ROSA26R* double transgenic FF3 (**a**), FF9 (**b**) and FM3 (**c**) embryos. X-gal staining is present both in the rostral and caudal *raphe* and absent in the r4-derived territory with a comparable pattern among the three distinct founders. r4: rhombomere 4. Scale bar: 500 µm.
(TIF)

Figure S3 ***Pet1_210_-Cre*** **somatic recombination mirrors** ***Pet1*** **spatio-temporal expression in the serotonergic system.** Representative sagittal 12.5 dpc, and coronal 15.5 dpc, P10 and P30 *Pet1_210_-Cre/ROSA26R* double transgenic (**a**, **d**, **g**, **j**) or wild-type (**b–c**, **e–f,** **h–i**, **k–l**) brain sections stained with X-gal chromogenic reaction or hybridized with a *Pet1* (**b**, **e**, **h**, **k**) or a *Tph2* (**c**, **f**, **i**, **l**) riboprobe, respectively. Note that at all the stages analysed β-galactosidase activity parallels with both *Pet1* and *Tph2* expression. Scale bar: 1 mm (**a**–**c**), 200 µm (**d**–**i**), 150 µm (**j**–**l**).
(TIF)

Figure S4 **Cre-mediated recombination outside the** ***raphe*** **in** ***Pet1_210_-Cre*** **mouse line selectively occurs in pancreas and kidney.** X-gal staining performed on whole-mount tissues from *Pet1_210_-Cre/ROSA26R* (**a, b, c, d, e, f, g, h, i, j, k, l**) and *ROSA26R* (**a’, b’, c’, d’, e’, f’, g’, h’, i’, j’, k’, l’**) animals at P 0.5 (**a–f’**) or adult (**g–l’**), showing the early post-natal and terminal distribution of *Pet1*-expressing cell progeny both in the brain and in peripheral organs. Cre-mediated recombination occurs specifically in hindbrain (**a**), pancreas (**b**) and kidneys (**c**) of P 0.5 double transgenic pups, and it is confined to the mature *raphe* system (**g, g’**), pancreatic beta cells (**h, h’**) and renal UB-derived collecting ducts and ureter (**i, i’**) in adults. Evidence of Cre-mediated recombination was undetectable in whole-mount specimens and on sections of adrenal glands (arrow in c, insets in i and i’, respectively). No reporter expression is present either in heart (**d, j**), spleen (**e, k**) or liver (**f, l**) of *Pet1_210_-Cre/ROSA26R*, or in organs from *ROSA26R* mice (**a’, b’, c’, d’, e’, f’, g’, h’, i’, j’, k’, l’**). Scale bar: 1.7 mm (**g-g’, i-i’, j-j’, k-k’, l-l’**), 1.5 mm (**a-a’**), 1 mm (**b-b’, c-c’, f-f’, inset in i-i’**), 750 µm (**d-d’, e-e’, h-h’**).
(TIF)

Figure S5 ***Pet1*** **expression in the developing pancreas correlates with the reporter distribution in** ***Pet1_210_-Cre/ROSA26YFP*** **mouse line.** Images of serial coronal sections at the level of pancreas of *Pet1_210_-Cre/ROSA26YFP* 15.5 dpc embryos hybridized with *Pet1* (**a**), *YFP* (**b**), *Nkx2.2* (**c**), *Tph1* (**d**) and *Tph2* (**e**) riboprobes. *Pet1* expression correlates with the expression of both *YFP* and *Nkx2.2,* while neither *Tph2* nor *Tph1* expression is detected in the pancreas at this stage. Scale bar: 300 µm (**a–e**).
(TIF)
