# Supplementary figures and images for: Generation of Pet1210-Cre Transgenic Mouse Line Reveals Non-Serotonergic Expression Domains of Pet1 Both in CNS and Periphery
Source: PLoS One. 2014 Aug 6;9(8):e104318. doi: 10.1371/journal.pone.0104318 (PMC4123907; doi:10.1371/journal.pone.0104318)

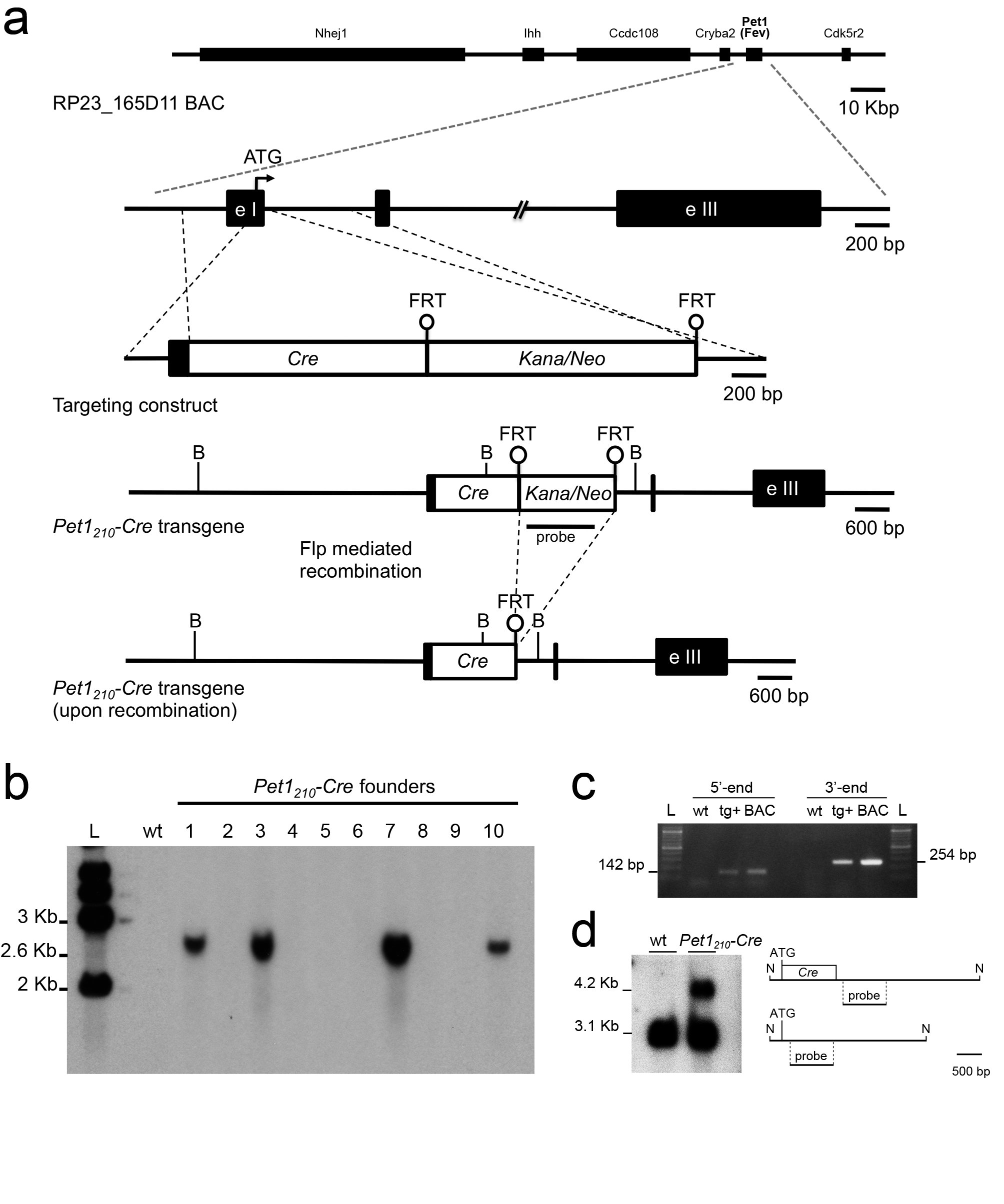

Supplement: Figure S1 — Pet1210-Cre allele generation. Diagram showing the wild-type Pet1 genomic locus contained within the RP23_165D11 BAC clone (a), the targeting vector for the homologous recombination in E. coli DY380 cells and the resulting Pet1210-Cre transgenic allele before and following Flp-mediated recombination. (b) Southern Blot analysis performed on genomic DNA obtained from Pet1210-Cre founders after BamHI digestion and hybridization with a probe against Kana/Neo cassette. Among the founders analysed, 4 presented the expected band at 2.6 kb. Founder-male 3 (FM3, lane 1), founder-female 3 (FF3, lane 7) and founder-female 9 (FF9, lane 10) showed germline transmission. (c) A 142 bp fragment at the 5′-end and a 254 bp fragment at the 3′-end of the transgene were amplified from the Pet1210-Cre FF9 founder genomic DNA and from the pBACe3.6 backbone assessing the integrity of the BAC transgene within mouse genomic DNA. (d) Southern Blot analysis performed on genomic DNA obtained from Pet1210-Cre FF9 founder after NotI digestion and hybridization with a probe capable to discriminate wt Pet1 locus vs Pet1210-Cre transgene. A clear lower hybridization intensity of the Pet1210-Cre transgene (4.2 kb) as compared to the Pet1 wt allele (3.1 kb) confirms the presence of a single copy of the transgene. L: ladder; wt: wild-type genomic DNA; tg+: Pet1210-Cre FF9 derived genomic DNA; BAC: RP23_165D11 BAC clone. (TIF) [file pone.0104318.s001.tif]

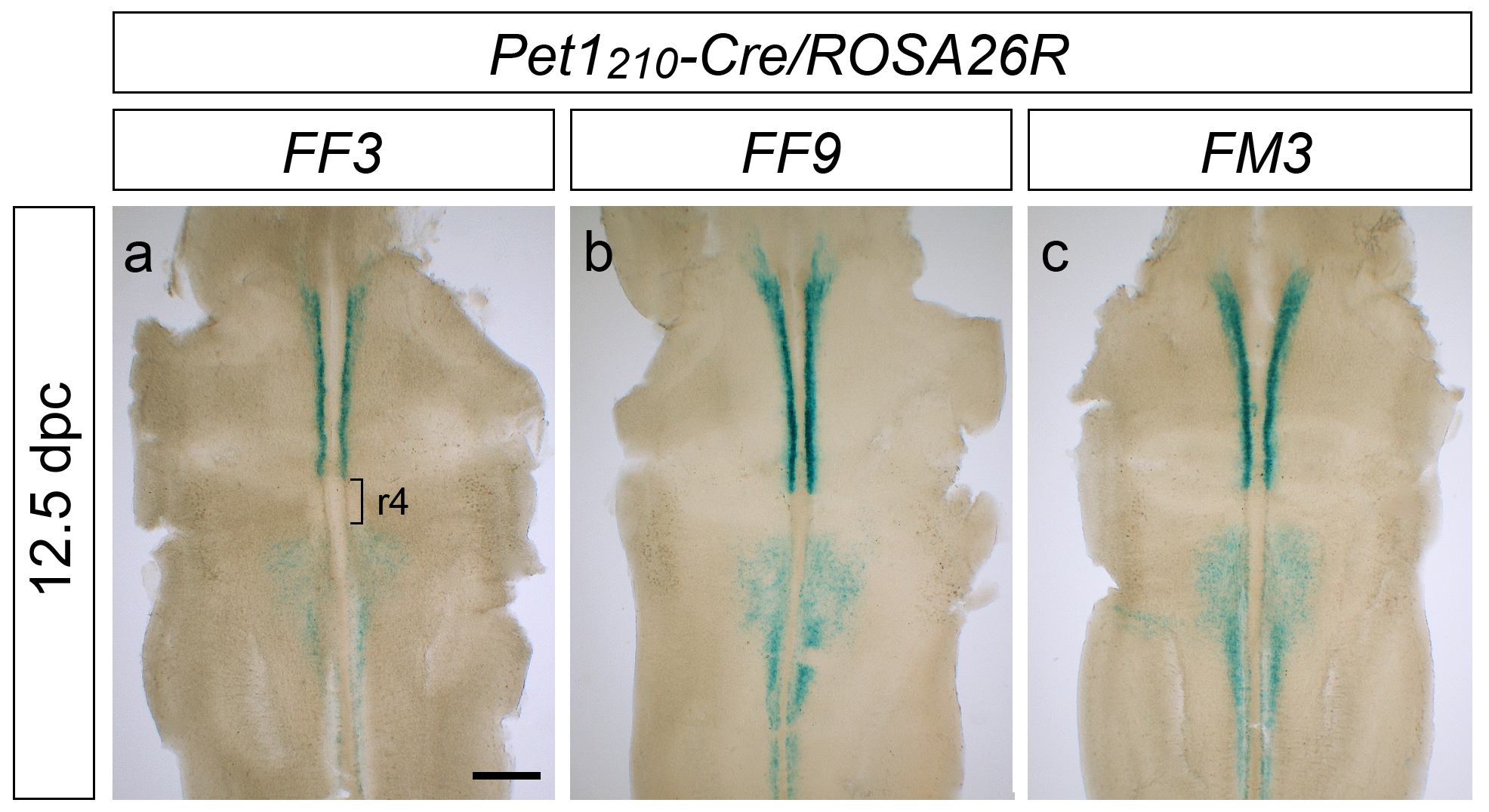

Supplement: Figure S2 — Pet1 drives Cre-mediated recombination in the serotonergic domains of the three Pet1210-Cre founders. Flat mount preparations of 12.5 dpc hindbrains obtained from Pet1210-Cre/ROSA26R double transgenic FF3 (a), FF9 (b) and FM3 (c) embryos. X-gal staining is present both in the rostral and caudal raphe and absent in the r4-derived territory with a comparable pattern among the three distinct founders. r4: rhombomere 4. Scale bar: 500 µm. (TIF) [file pone.0104318.s002.tif]

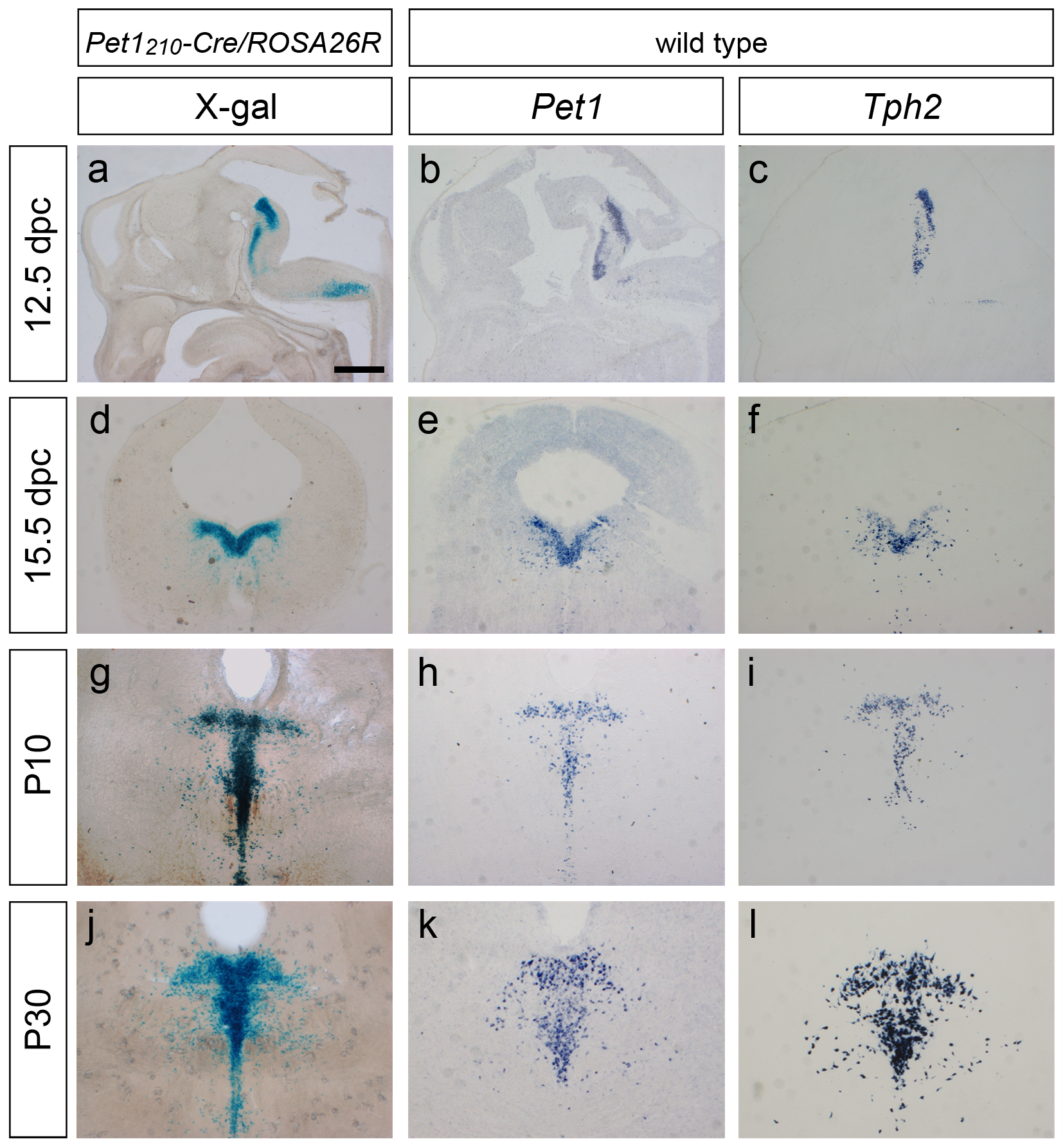

Supplement: Figure S3 — Pet1210-Cre somatic recombination mirrors Pet1 spatio-temporal expression in the serotonergic system. Representative sagittal 12.5 dpc, and coronal 15.5 dpc, P10 and P30 Pet1210-Cre/ROSA26R double transgenic (a, d, g, j) or wild-type (b–c, e–f, h–i, k–l) brain sections stained with X-gal chromogenic reaction or hybridized with a Pet1 (b, e, h, k) or a Tph2 (c, f, i, l) riboprobe, respectively. Note that at all the stages analysed β-galactosidase activity parallels with both Pet1 and Tph2 expression. Scale bar: 1 mm (a–c), 200 µm (d–i), 150 µm (j–l). (TIF) [file pone.0104318.s003.tif]

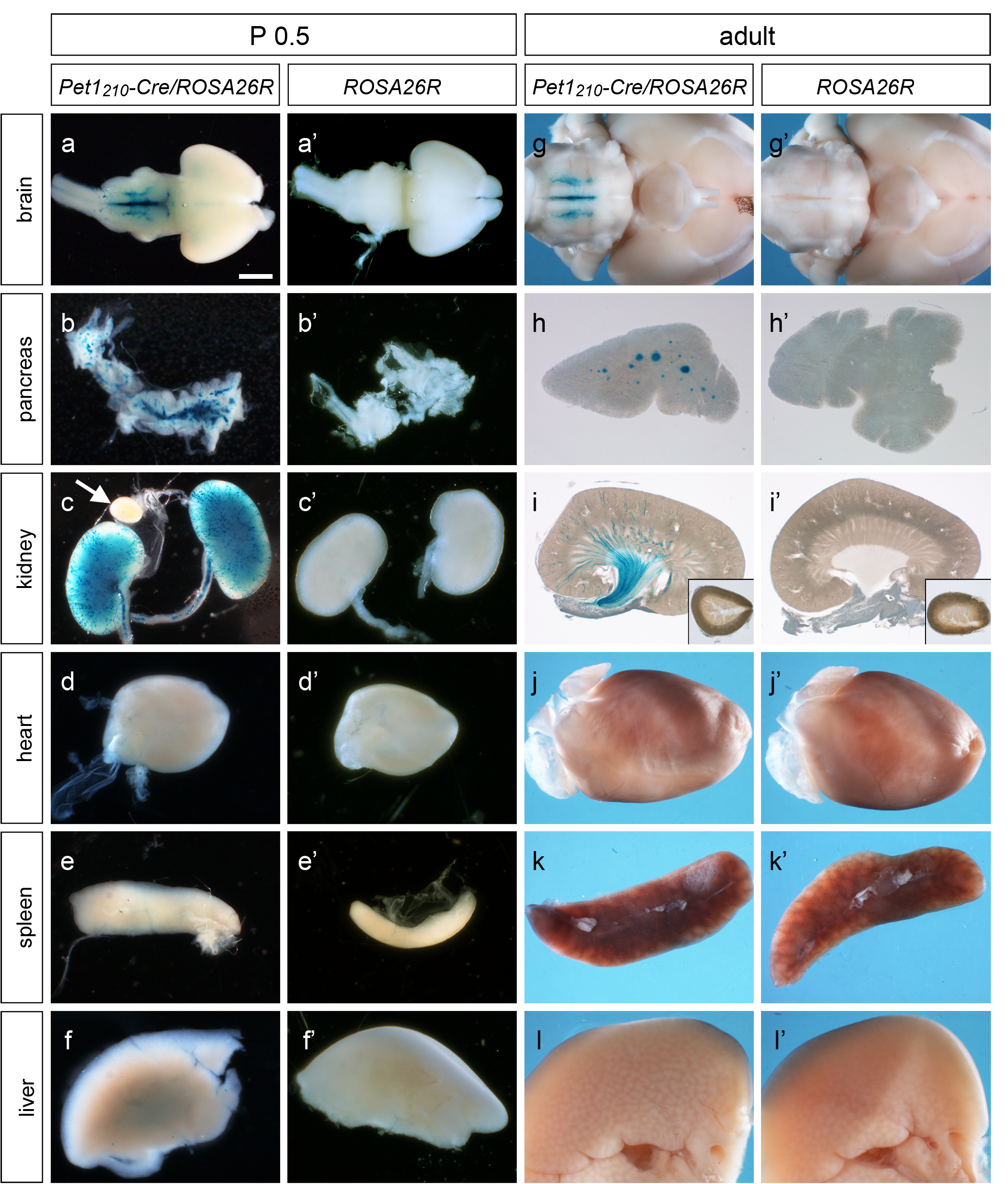

Supplement: Figure S4 — Cre-mediated recombination outside the raphe in Pet1210-Cre mouse line selectively occurs in pancreas and kidney. X-gal staining performed on whole-mount tissues from Pet1210-Cre/ROSA26R (a, b, c, d, e, f, g, h, i, j, k, l) and ROSA26R (a’, b’, c’, d’, e’, f’, g’, h’, i’, j’, k’, l’) animals at P 0.5 (a–f’) or adult (g–l’), showing the early post-natal and terminal distribution of Pet1-expressing cell progeny both in the brain and in peripheral organs. Cre-mediated recombination occurs specifically in hindbrain (a), pancreas (b) and kidneys (c) of P 0.5 double transgenic pups, and it is confined to the mature raphe system (g, g’), pancreatic beta cells (h, h’) and renal UB-derived collecting ducts and ureter (i, i’) in adults. Evidence of Cre-mediated recombination was undetectable in whole-mount specimens and on sections of adrenal glands (arrow in c, insets in i and i’, respectively). No reporter expression is present either in heart (d, j), spleen (e, k) or liver (f, l) of Pet1210-Cre/ROSA26R, or in organs from ROSA26R mice (a’, b’, c’, d’, e’, f’, g’, h’, i’, j’, k’, l’). Scale bar: 1.7 mm (g-g’, i-i’, j-j’, k-k’, l-l’), 1.5 mm (a-a’), 1 mm (b-b’, c-c’, f-f’, inset in i-i’), 750 µm (d-d’, e-e’, h-h’). (TIF) [file pone.0104318.s004.tif]

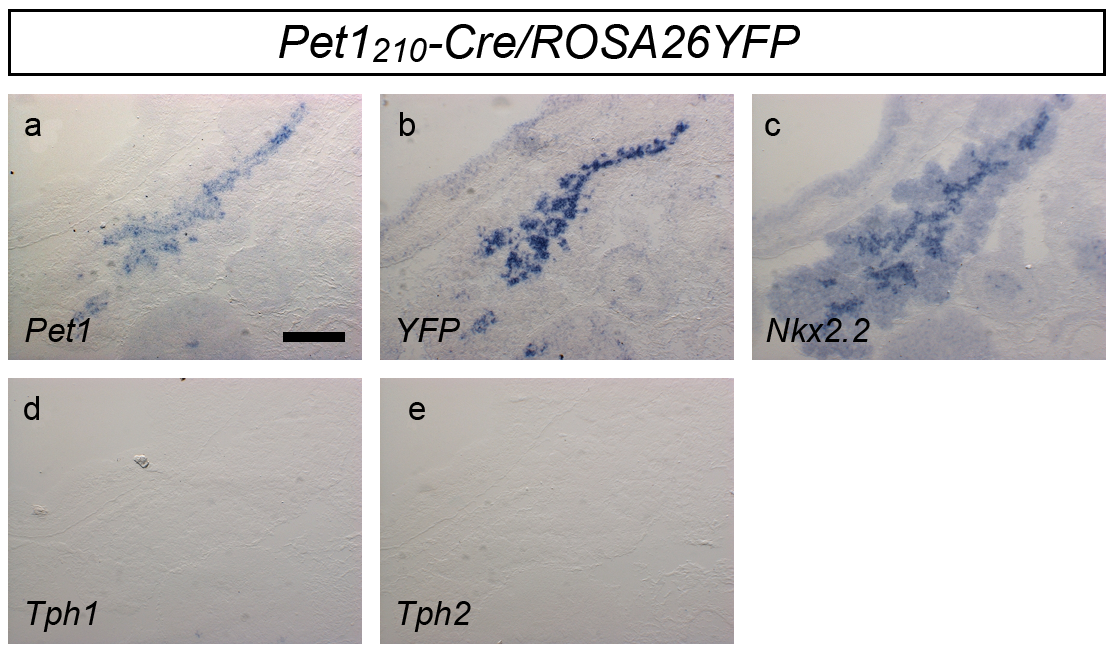

Supplement: Figure S5 — Pet1 expression in the developing pancreas correlates with the reporter distribution in Pet1210-Cre/ROSA26YFP mouse line. Images of serial coronal sections at the level of pancreas of Pet1210-Cre/ROSA26YFP 15.5 dpc embryos hybridized with Pet1 (a), YFP (b), Nkx2.2 (c), Tph1 (d) and Tph2 (e) riboprobes. Pet1 expression correlates with the expression of both YFP and Nkx2.2, while neither Tph2 nor Tph1 expression is detected in the pancreas at this stage. Scale bar: 300 µm (a–e). (TIF) [file pone.0104318.s005.tif]
